# Effect of roasting on the chemical and lipid composition of pine nuts in two regions in Russia

**DOI:** 10.1016/j.heliyon.2024.e34576

**Published:** 2024-07-14

**Authors:** Olga Teneva, Zhana Petkova, Nikolay Toshev, Nikolay Solakov, Kamelia Loginovska, Yurii Platov

**Affiliations:** a University of Plovdiv ‘Paisii Hilendarski’, Department of Chemical Technology, 24 Tzar Assen Street, Plovdiv, 4000, Bulgaria; b Medical University of Plovdiv, Faculty of Pharmacy, Department of Bioorganic Chemistry, 15-A “Vasil Aprilov” blvd, Plovdiv, 4002, Bulgaria; c Agricultural Academy, Institute of Cryobiology and Food Technology, Department of Biological Active Substance Technology, 1407, Sofia, bul. "Cherni Vrah" 53, Bulgaria; d Plekhanov Russian University of Economics, Faculty of Trade Economics and Commodity Science, Department of Commodity Science, Stremyanny lane 36, RU 115054, Moscow, Russia

**Keywords:** Pine nuts, Fatty acids, Roasting, Lipids, Bioactive components

## Abstract

The impact of thermal treatment for roasting on pine nuts from two geographical regions in Russia (Vladivostok and Baikal) was studied. They were roasted at 180 °C for 20 min in an oven. The aim was to establish the changes that occurred in the chemical (proteins, lipids, carbohydrates, ash, fibers), lipid composition (fatty acids, tocopherols, sterols, phospholipids), and physicochemical characteristics (peroxide value, acid value, iodine value, conjugated dienes and trienes) after roasting of the pine nuts. The results showed that the composition of the raw and roasted nuts differed (p < 0.05). Significant differences (p < 0.05) were observed in the oxidative stability of raw and roasted nuts from Baikal and, the content of unsaponifiable substances and sterols in the nut oil from Vladivostok. The roasting process influences the isolated oils from pine nuts as the chemical and lipid composition changes of oils from Baikal are significant (p < 0.05). Both raw and roasted pine nuts have high energy value, protein content, and good health benefits and can be consumed either way. Overall, it was established that the roasting of pine nuts had little impact on their lipid composition; the nuts could also be a source of lipid-soluble bioactive components and might have health–promoting effects.

## Highlights

• Roasting impacts the quality of pine nuts.
• Changes in chemical composition of pine nuts during roasting.
• Influence of roasting on the lipid composition of the nuts.
• Differences in physicochemical characteristics of the oils after roasting.

## 1 Introduction

Edible nuts are helpful because of the nutritional and health attributes they may exert - they are rich sources of lipids and proteins, vitamins, and minerals. According to the Food and Agriculture Organization, pine nuts are edible and have high nutritional value and health benefits [1,2]. The genus Pinus (Pinaceae family) includes about 250 species and is typically found in the Mediterranean region but it is common in other parts of the world, such as Asian regions. China, Russia (Siberia), Korea, and Pakistan are the leading countries for exporting [3,4]. Approximately 20,000 tons of pine nuts were produced in the world for the period 2017–2018, with China as the leading producer (39 %), followed by North Korea, Pakistan, and Afghanistan (with 13 % each) [5]. The total production of tree nuts in Russia in 2010 was 17,930 metric tons (MT), and those of pine nuts was about 4500 MT, which are produced mainly for export. These nuts are widespread in Russia: Siberia and Far East regions. The yield of the nuts depends on the harvesting areas and the conditions of the weather [6].

Pine nuts can be used in many areas as they are most important for human health. They are used mainly in the food industry. They can be consumed either raw or roasted. Pine nuts are rich in protein and lipids (unsaturated fatty acids in their composition can be up to 85 %). They are an excellent source of glyceride oil and lipid-soluble biologically active components (fatty acids, tocopherols, sterols, phospholipids). Consumption of raw nuts with high quantities of unsaturated fatty acids and antioxidants improves health status as it reduces the risk of cardiovascular diseases by decreasing serum levels of low-density lipoprotein - (LDL-) cholesterol and risk of developing type II diabetes and strengthens the immune system [[7], [8], [9]]. On the other hand, the same fatty acids can lead to lower oxidative stability of the oils because they are easily influenced by atmospheric conditions (high temperature, oxygen, light, etc.), which causes lipid oxidation. The composition of the pine nuts is also influenced at some extent on their origin, growing conditions and different species.

Thermal treatment is one of the most popular ways to process the raw nuts. It is well established that some characteristics of the nuts are improved after roasting, for example sensorial acceptance, several antinutritional compounds are reduced, *etc*. From this point of view, roasting influences the chemical and lipid composition of the nuts and their accompanying health benefits [10,11]. According to Hosseini et al. [12] the roasting process influences the protein content by decreasing its content. On the other hand, the oil yield of the pine nuts was observed to increase with the prolonging of the roasting time [13]. However, the levels of some of the thermoliable components in the pine nut oils can drastically decrease, such as tocopherols, sterols, unsaturated fatty acids, *etc*. [13]. What is more, the thermal treatment can also deteriorate the quality of the nuts: decreasing the amount of the bioactive components and increasing the levels of the physicochemical characteristics of the glyceride oils.

However, in depth studies on the impact of roasting on the chemical and lipid composition of nuts from two species of pines originating from different regions in Russia are very limited. Therefore, the aims of the study were to establish the differences in the chemical and lipid composition of two different raw pine nuts from two Russian regions: *Pinus sibirica* from Baikal and *Pinus koraiensis* from Vladivostok) as well as revealing the influence of roasting on their composition.

## 2 Materials and methods

### 2.1 Materials

The study was carried out with pine nuts from two regions in Russia: *Pinus sibirica* from Baikal and *Pinus koraiensis* from Vladivostok. The pine cones were collected in October 2022. The mature pine cones with nuts were gathered from the ground in the taiga in the vicinity of the urban village of Kavalerovo (coordinates 44.267653, 135.05726, which are linked to the world system of geodetic parameters WGS84), located 285 km to the north-west of Vladivostok (regional center of Primorsky Krai, Russian Federation) and the other part of the samples were collected from a location near Listvyanka, Irkutsky District, lake Baikal, Russia (51.853784, 104.892697). The nuts were harvested from fifteen trees located in 25 m in distance from each other. The collected cones were packed in bags and taken to a warehouse where peeling was carried out. Then, the cones were broken, the seeds removed, and the pine nuts crashed to remove the shell. After shelling, the pine nuts were air-dried and samples of 1000 g (from each region) were randomly selected from a batch of pine nuts.

The analyses were conducted before and after the heat treatment process. 500 g of nuts were subjected to roasting at 180 °C for 20 min in a pre-heated oven without air circulation (Eldom 203 VFEN, 38 L, Varna, Bulgaria) and the nuts were stirred in the interval of 3 min.

### 2.2 Chemical composition

Crude fibers, moisture, and ash content were determined according to AOAC [14]. Total protein content was determined as follows: 1 g of crashed nuts were mineralized with a mixture of sulfuric acid and hydrogen peroxide for 35 min at 420 °C. After that the solution was destilled with water solution of sodium hydroxide and then the sample was titrated with 0.1 N sulfuric acid. The total protein content was calculated on the basis of the % of nitrogen [14]. The content of carbohydrates was calculated as follow [1]:

100 – (sum of the contents, in %, of proteins, lipids, ash and moisture)

Soxhlet extraction with n-hexane was used to separate the glyceride oil from the nuts. The extraction procedure was carried out with 50 g of pine nuts [15]. The energy value of the seeds was determined using the formula [1]:

EV = C × 4 + L × 9 + P × 4 (kcal/100g)

where EV is the energy value, C is the total carbohydrates (%), L is the total lipids (%), and P is the total proteins (%).

### 2.3 Physicochemical characteristics of glyceride oil

The physicochemical properties of oils were determined using the following procedures: peroxide value**,** acid value, and iodine value [[16], [17], [18]]. The absorbance at 232 and 270 nm (A_232_ and A_270_) was determined using a spectrophotometer Boeco S26 (Hamburg, Germany) after diluting the oil in isooctane (1/100, v/v) [19]. The equipment used to determine oxidative stability was Rancimat 679 (Metrohm, Herisau, Switzerland) [20]. The measurement conditions were 100 °C and an air flow rate of 20 L/h.

### 2.4 Free radicals scavenging activity (DPPH)

The DPPH (2,2- diphenyl-1-1 picrylhydrazyl) was used to estimate the antioxidant activity of pine nut oil [21]. 25 mg of the oil were dissolved in 2.5 ml solvent (80 % Acetone and 20 % Methanol). After mixing with the solvent, the sample was shaken with a vortex for 5 min 1 ml of this solution was added to 1 ml DPPH (0.1 M), and the sample was left for 30 min in a dark place. After that, the measurement was carried out spectrophotometrically at 517 nm on Boeco S26 (Hamburg, Germany). The results were expressed as μg Ascorbyl palmitate equivalent/g oil.

### 2.5 Fatty acid composition

The composition of fatty acids in the glyceride oil was determined using gas chromatography (GC) [22]. Fatty acid methyl esters (FAMEs) were obtained by transesterification of the oil with sulfuric acid in methanol [23]. Determination was performed on an Agilent 8860 gas chromatograph (Santa Clara, California, USA) equipped with a capillary column DB FastFAME (30 m 0.25 mm 0.25 mm (film thickness)) and a flame ionization detector (FID). The column temperature was from 70 °C (1 min), at 6 °C/min to 180 °C, and at 5 °C/min to 250 °C; the injector and detector temperatures were 270 °C and 300 °C respectively; the carrier gas was nitrogen. Identification was carried out by comparison of the retention times of a standard mixture of FAME (Supelco, Sigma-Aldrich Chemical Co. (St. Louis, MO, USA) with a purity ∼99 %).

### 2.6 Sterols

Glyceride oil was saponified, and the unsaponifiable fraction was extracted with n-hexane [24]. Total sterols were measured spectrophotometrically at 597 nm on Boeco S26 (Hamburg, Germany) after the isolation of sterols from the unsaponifiable matter by TLC [25]. Sterol composition was determined on an HP 5890 gas chromatograph (California, USA) with DB 5 capillary column (25 m 0.25 mm 0.25 mm (film thickness)) and FID. The temperature gradient was from 90 °C (3 min) up to 290 °C at a rate of change of 15 °C/min and then up to 310 °C at a rate of 4 °C/min (10 min); detector temperature: 320 °C; injector temperature: 300 °C and carrier gas was hydrogen. Identification was confirmed by comparison of retention times with a standard mixture of sterols [26].

### 2.7 Tocopherols

The qualitative and quantitative composition of tocopherols was determined directly in the oil by high-performance liquid chromatography (HPLC) on a Merck – Hitachi equipped with Nucleosil Si 50-5 column (250 × 4 mm, particle size: 5 mm), fluorescent detection at 290 nm excitement and 330 nm emission as the fluorescent detector was Merck – Hitachi F 1000. The operating conditions were a mobile phase of hexane:dioxane, 96:4 (v/v), and a flow rate of 1 mL/min [27].

### 2.8 Phospholipids

Phospholipids were isolated from the nuts using extraction with a mixture of chloroform and methanol (2:1, v/v) [28]. The individual phospholipids were determined by two-dimensional thin-layer chromatography (TLC) [29]. Identification was performed by comparing the R_f_ values with standards. The spots of phospholipids were scrapped and mineralized with a mixture of perchloric and sulfuric acid, 1:1 (v/v), and the quantification was performed spectrophotometrically at 700 nm on Boeco S26 (Hamburg, Germany) [30].

For the calculation of the phosphorus content of the samples was prepared a stock solution of KH_2_PO_4_ in concentration of 100 μg/mL. Then, a standard curve was constructed by using the stock solution in concentration between 10 and 100 μg/mL. The phospholipid content in the sample was calculated as a percentage of the phosphorus:

$$X=\frac{f\times A}{m}\times 100$$

Where: X is the phosphorus content of the sample, %; f – is the calibration factor; A is absorbance measured.

### 2.9 Reagents

All reagents and solvents were of analytical grade. They are used without additional purification. Reference tocopherol (α-, β-, γ- and δ-tocopherol with purity ≥98 %) were purchased from Merck (Darmstadt, Germany). Individual sterols: cholesterol was purchased from Acros organics (New Jersey, USA), stigmasterol (with purity ∼ 95 %) was purchased from Sigma Aldrich (St. Louis, MO, USA) and β-sitosterol (with ca 10 % campesterol, ca 75 % β-sitosterol) was purchased from Acros organics (New Jersey, USA). Reference phospholipids (purity ≥97 %) were purchased from Fluka (Chemie GmbH, Buchs, Switzerland*).* Fatty acid methyl esters were purchased from Supelco, Sigma-Aldrich Chemical Co. (St. Louis, MO, USA) with purity ∼99 %.

### 2.10 Statistics

All measurements were performed in triplicate (n = 3), and the results were presented as mean value ± standard deviation (SD). The significant differences in the mean values were established by a post-hoc test Duncan, at p < 0.05.

## 3 Results and discussion

### 3.1 Chemical composition

Raw and roasted pine nuts were investigated concerning glyceride oil, protein, carbohydrates, fibers, ash, moisture content, and energy value. The results are presented in Table 1.

**Table 1:** Chemical composition of raw and roasted pine nuts.

	Baikal		Vladivostok	
Compounds, %	Raw	Roasted	Raw	Roasted
Glyceride oil	49.97 ± 0.34^а^	53.13 ± 0.48^b^	64.41 ± 0.36^c^	65.20 ± 0.42^d^
Protein	14.80 ± 0.12^а^	15.30 ± 0.08^b^	14.88 ± 0.05^а^	14.81 ± 0.04^а^
Carbohydrates	28.99 ± 0.15^а^	23.81 ± 0.19^b^	15.32 ± 0.14^c^	16.64 ± 0.17^d^
Crude fiber	7.61 ± 0.05^а^	5.53 ± 0.04^b^	10.60 ± 0.07^c^	13.20 ± 0.12^d^
Ash	2.51 ± 0.08^bc^	2.70 ± 0.05^c^	1.93 ± 0.19^а^	2.38 ± 0.08^b^
Moisture	3.74 ± 0.17^a^	2.09 ± 0.05^b^	3.46 ± 0.1^c^	0.97 ± 0.05^d^
Energy value, kcal/100g	625	662	700	713

* Different small letters in a row depict significant differences in the results (p < 0.05).

All the results were performed in triplicates (n = 3).

The chemical composition of raw pine nuts from both geographical regions, Baikal and Vladivostok, was determined by their content of glyceride oil, protein, carbohydrates, crude fiber, ash, moisture, and energy value. Significant differences (p < 0.05) in the content for some components were established (glyceride oil, carbohydrates, crude fiber, ash, and moisture). The amount of glyceride oil and crude fiber was higher in pine nuts from Vladivostok, while the carbohydrate and ash content is higher in the nuts from Baikal. On the other hand, the protein content was similar in both samples.

Notable differences were observed in glyceride oil content after roasting the nuts from both Russian regions (p < 0.05). The amount of oil was higher in the roasted ones. The possible explanation is the water evaporation during the roasting treatment (Baikal from 3.74 to 2.09 % and Vladivostok from 3.46 to 0.97 %). The content of carbohydrates and crude fiber decreased after roasting the nuts from Baikal, while in those from Vladivostok, the quantity of the same components increased. A significant increase (p < 0.05) in protein content after roasting Baikal nuts was observed, but the ones from Vladivostok did not influence it (p > 0.05).

The data for the oil content of the raw nuts from Baikal (49.97 %) was similar to Valero-Galvan et al. [31], where it was 44.9 % for pine nuts of *P. pinea*. According to Wolf & Bayard [32], it was 64.0 % for investigated *P. cembroides*, which was more similar to raw and roasted pine nuts from Vladivostok. The thermal treatment increases the oil yield for the roasted nuts from both regions, which agrees with An et al. [13] where the nuts are roasted at the same conditions (180 °C, 20 min).

Pine nuts are a good source of proteins. The protein content of raw and roasted pine nuts from Baikal and Vladivostok was about 15 %. The data about the protein content of analyzed raw nuts from Baikal and Vladivostok were significantly different compared to the data received from other authors. According to Zuleta et al. [33], it was 32.1–36.6 % for raw pine nuts from six countries. The protein content can probably be affected more by agro-climatic conditions than heat treatment. According to Hosseini et al. [12], the protein content decreased after roasting, while it remained similar for raw and roasted nuts from Vladivostok.

The examined nuts were rich in carbohydrates, too. The results for total carbohydrates of raw nuts from Vladivostok agree with the results obtained by previous authors, where the content is 13.9 % for nuts of *P. pinea* [34], while the amount in the nuts from Baikal is much higher.

The fibers are of great importance for the gastrointestinal tract. The nuts were rich in fibers, and because of that, they were very suitable for consumption, both raw and roasted. The fiber content of the raw nuts was 10.6 % (Vladivostok) and 7.6 % (Baikal). The results for raw nuts from Vladivostok were almost the same as the fiber content for pine nuts of *P. koraiensis* (10.8 %) reported by Lixia et al. [35]. According to Zuleta et al. [33], the values of fibers obtained from raw pine nuts from six countries were higher (between 9.8 and 14.6 %), corresponding with the geographic areas and agro-climatic conditions. In turn, the value of roasted nuts from Vladivostok (13.2 %) was higher than from Baikal (5.5 %).

Ash content was similar for all investigated nuts. It corresponded with the data for pine nuts of *P. koraiensis* (2.8 %) except for the raw nuts from Vladivostok, which had the lowest ash content. According to Nergiz & Dönmez [34], the ash content was about two times lower in the nuts of *P. pinea* (4.50 %).

There was scarce data regarding the moisture content in pine nuts. Nergiz & Dönmez [34] reported that pine nuts of *P. pinea* and *P. maximartinezii*, had about 5.0 % while *P. halepensis* and *P. pinaster* about 8.0 %. Due to the treatment's high temperature, the roasted pine nuts from Baikal and Vladivostok had less moisture than the raw ones.

Raw and roasted nuts are a good source of macronutrients – proteins, glyceride oils, and carbohydrates- and are characterized by high energy value. The energy of the nuts depends on the content of lipids, proteins, and carbohydrates [36]. Because of that, it is essential to monitor the changes in chemical composition after roasting. The energy value of the nuts from Baikal was 625 kcal/100g for raw and 662 kcal/100 g for roasted, while the energy value for pine nuts from Vladivostok was slightly higher.

### 3.2 Physicochemical characteristics

The results of the physicochemical characteristics of the raw and roasted pine nuts are presented in Table 2.

**Table 2:** Physicochemical characteristics, A_232_, A_270_ and oxidative stability of raw and roasted pine nuts.

Physicochemical characteristics	Baikal		Vladivostok	
	Raw	Roasted	Raw	Roasted
Peroxide value, meqO_2_/kg	4.49 ± 0.18^а^	6.91 ± 0.10^b^	4.70 ± 0.12^a^	6.80 ± 0.20^b^
Acid value, mgKOH/g	4.09 ± 0.05^a^	4.15 ± 0.10^a^	0.92 ± 0.08^b^	0.95 ± 0.05^b^
Iodine value, g I_2_/100g	152.7 ± 1.3^a^	155.2 ± 1.5^b^	132.5 ± 1.2^c^	127.3 ± 1.0^d^
A_232_	0.404 ± 0.024^ab^	0.525 ± 0.043^c^	0.361 ± 0.033^a^	0.466 ± 0.053^bc^
A_270_	0.074 ± 0.006^a^	0.086 ± 0.002^b^	0.023 ± 0.004^c^	0.056 ± 0.005^d^
Oxidative stability, h	27.5 ± 0.4^c^	14.5 ± 0.2^b^	14.0 ± 0.2^ab^	13.9 ± 0.3^a^

* Different small letters in a row depict significant differences in the results (p < 0.05).

All the results were performed in triplicates (n = 3).

Peroxide value indicates the amount of hydroperoxides contained in the oil. These compounds accumulate during lipid oxidation, and they are the first products to cause the deterioration of the quality of the oils. It depends on the number of peroxide values - the lower the value, the better the oil quality. Sometimes, the nuts can be stored a long time before the roasting process, which may increase the peroxide value. Furthermore, the storage conditions and the oil extraction method can affect the fatty acid composition of oils and their peroxide value.

According to Olatidoye et al. [37], roasting is a process that can lead to changes in many components – loss of substances such as volatile compounds, water vapor, degradation of polysaccharides, sugars, amino acids, and chlorogenic acids, which can form products of caramelization and condensation with an increase in organic acids and lipids. While roasting, the nuts from Baikal and Vladivostok formed free radicals, which formed hydroperoxides.

Reports by other authors found that the peroxide value of raw pine nut oil of *P. koraiensis* was 2.47 mmol/kg [35]. The peroxide value of the investigated oils from Baikal and Vladivostok increased after roasting from 4.5 to 6.9 meqO_2_/kg (Baikal) and from 4.7 to 6.8 meqO_2_/kg (Vladivostok) (p < 0.05). This heightening of the peroxide value of roasted pine nut oils can be explained by the absorption of oxygen, which increases the formation of peroxides due to the heating of the oil during the temperature treatment (180 °C). The results agreed with Hosseini et al. [12], who reported that the peroxide value was affected by roasting temperature. On the other hand, the peroxide value in a range lower than 10 meqO_2_/kg was noticed for fresh and healthy oils.

The acid value indicates the quantity of free fatty acids present in vegetable oils and provides information about the rate of hydrolysis. The acid value of the oils extracted from raw nuts from both regions increased insignificantly after the roasting process (p > 0.05). That means free fatty acids are released during heat treatment through enzymatic hydrolysis. The acid value for raw nut oil from Vladivostok (0.92 mgKOH/g) was close to that reported earlier by Lixia et al. [35] (0.51 mgKOH/g) and different for raw nut oil from Baikal (4.09 mgKOH/g).

The iodine value is an analytical characteristic of the oils, which presents the degree of unsaturation in oil components, which the uptake of halogen can determine. The iodine value of pine nut oils from Baikal and Vladivostok was 152.7 g I_2_/100g and 132.5 g I_2_/100g, respectively. The results were close to the data from Lixia et al. [35], which was 138.89 g I_2_/100g. It is established that quantities of C18:2 (linoleic) and C 18:3 (Pinolenic) fatty acids of pine nut oil from roasted nuts from Vladivostok decreased due to heat treatment, and the iodine value decreased because of processes of intensive thermo oxidation. The tendency of analyzed oils from Baikal was the opposite – the rate of iodine value increased for roasted compared with raw pine nut oils. On the one hand, notable differences relative to examined raw and roasted nuts from Baikal and Vladivostok were not found (p > 0.05). However, on the other hand, oxidative stability is related to the degree of unsaturation, and the iodine value provides an estimation regarding it. The greater the iodine value, the more unsaturation and the higher the susceptibility to oxidation.

The absorbance of the oils diluted in isooctane at 232 and 270 nm (A_232_ and A_270_) gives the rate of accumulation of conjugated dienes and trienes, and these characteristics can be used as indicators for increasing the presence of hydroperoxides in the lipids. The glyceride oils isolated from the raw nuts were distinguished with lower absorbance for A_232_ and A_270_ (0.404 and 0.074 in the oil from Baikal; 0.361 and 0.023 in the oil from Vladivostok). After the roasting of the nuts, there was a slight increase in these characteristics, which indicated the accumulation of hydroxides in the lipids (p < 0.05). Despite that, even the values for A_232_ of the roasted nut oils were below the recommended ones for evaluation of the rate of oxidation – up to 0.60 for fresh oils, 0.70–0.85 for slightly oxidized oils, 1.00–1.25 for oxidized oils, and over 1.25 for extremely oxidized oils [38]. The oxidative stability of the oils was measured using the Rancimat test, and the induction time was determined. The oxidative stability is related to fatty acid composition, the availability of tocopherols, etc., which are good natural antioxidants, and the presence of synergists. When the oils have primarily polyunsaturated fatty acids, they can be oxidized more quickly, and their peroxide value rises. The oxidative stability usually decreases during heat treatment because of accelerated lipid oxidation. The results of the analyzed pine nut oil from Baikal agreed with this statement, as the oxidative stability of pine oil from raw nuts was about two times higher than that of roasted ones. The moisture level of roasted pine nuts and the higher quantity of unsaturated fatty acids are possibly the main reasons that lead to oxidation and degradation. The investigated pine oils from Vladivostok (raw and roasted) had equal oxidative stability, i.e., the high temperature had not influenced the oil quality. This was related to the amount of tocopherols as primary natural antioxidants in fats and oils, which were almost unaffected in the analyzed oils during heat treatment.

The antioxidant activity of raw and roasted nuts from Vladivostok using the DPPH method was also established. The determined values for raw nut oil were 86.1 μg Ascorbyl palmitate equivalent/g and 46.1 μg Ascorbyl palmitate equivalent/g for the oil of roasted nuts. In the pine nuts from Baikal was determined also higher antioxidant activity in the raw samples (95.1 μg Ascorbyl palmitate equivalent/g) than of the roasted ones (55.2 μg Ascorbyl palmitate equivalent/g). A significant decrease in antioxidant activity was observed after thermal treatment of the nuts and oils (p < 0.05). Completely different results for the antioxidant capacity of the nut oils using the DPPH method (expressed as IC_50_) were reported by An et al. [13], who established that the highest level of antioxidant activity was observed in the oil after thermal treatment at 180 °C for 30 min.

### 3.3 Biologically active components

The biologically active components of pine nuts are fatty acids, unsaponifiable compounds, sterols, phospholipids, and tocopherols. They have good health benefits but can be affected by heat treatment. All results about the content of biologically active components of analyzed pine oils are presented in Table 3.

**Table 3:** Content of biologically active components of raw and roasted pine nut oils.

Components	Baikal		Vladivostok	
	Raw	Roasted	Raw	Roasted
Unsaponifiable matter, %				
-in the oil	3.23 ± 0.13^b^	2.96 ± 0.08^a^	3.43 ± 0.10^b^	17.97 ± 0.15^c^
-in the nuts	1.61 ± 0.06^a^	1.66 ± 0.04^a^	2.21 ± 0.06^b^	11.72 ± 0.09^c^
Sterols, % -in the unsaponifiable matter	10.53 ± 0.55	10.47 ± 0.35	13.41 ± 0.74	2.45 ± 0.12
-in the oil	0.34 ± 0.05^a^	0.31 ± 0.04^a^	0.46 ± 0.06^b^	0.44 ± 0.05^b^
-in the nuts	0.17 ± 0.03^a^	0.17 ± 0.02^a^	0.30 ± 0.04^b^	0.29 ± 0.03^b^
Phospholipids, %				
-in the oil	1.40 ± 0.12^b^	1.10 ± 0.14^a^	2.10 ± 0.18^c^	2.29 ± 0.08^c^
-in the nuts	0.70 ± 0.06^a^	0.62 ± 0.08^a^	1.35 ± 0.12^b^	1.49 ± 0.05^b^
Tocopherols, mg/kg				
-in the oil	271 ± 16^b^	311 ± 10^c^	110±8^a^	114 ± 12^a^
-in the nuts	135±8^b^	175±6^c^	71±5^a^	74±8^a^

* Different small letters in a row depict significant differences in the results (p < 0.05).

All the results were performed in triplicates (n = 3).

In previous studies, data about unsaponifiable matter content in pine nut oil could not be found. The amount of unsaponifiable matter in the nut oil from Baikal is 3.23 % (raw) and 2.96 % (roasted), respectively. On the other hand, the results observed for analyzed oils from Vladivostok are 3.43 % (raw) and 17.97 % (roasted). Similar results were reported by El-Labban [39] who monitored the impact of the roasting process on flaxseeds. The author established that the content of the unsaponifiable matter and total sterols slightly increased during roasting and the possible reason for that was the damage of the cell structure. All amounts are higher than reported in Codex Stan 19 [40] for other vegetable oils where the quantity of unsaponifiable matter is 1.0 % (for peanut oil), 2.0 % (rapeseed oil), 1.5 % (soybean oil) etc.

A major part of the unsaponifiable matter consists of sterols. There is no significant difference determined between sterol content in the oil from raw nuts (0.34 %) and roasted nuts (0.31 %) from Baikal, as well as in the oil from raw pine nuts (0.46 %) and roasted pine nuts (0.44 %) from Vladivostok. The process of heat treatment does not influence the quantities of the sterols.

The total content of phospholipids in all analyzed pine nut oils is established. The quantities are similar for oils from Vladivostok – raw (2.1 %) and roasted (2.29 %), while some differences are found for oils from Baikal – raw (1.4 %) and roasted (1.1 %).

The total tocopherol content of the examined oils is 271 mg/kg for raw and 311 mg/kg for roasted (Baikal). The data about the tocopherol content of oils from Vladivostok is notably different than that from Baikal. The analyzed oils from raw and roasted nuts from Vladivostok are similar - 110 mg/kg for raw and 114 mg/kg for roasted (p > 0.05). The possible reason for the slightly increase of the tocopherol content in the oils from the Baikal nuts was their microstructures that might lead to a different behavior of this compounds during roasting. Such a conclusion was reported by Durmaz & Gökmen [41]. The results for tocopherol content in oils significantly differ from the data presented by An et al. [13], where the levels of tocopherols decrease at high temperatures. The same tendency is observed for sunflower oil [42].

Changes in the fatty acid composition after roasting the pine nuts are shown in Table 4.

**Table 4:** Fatty acid composition of raw and roasted pine nut oils.

Fatty acids, %		Baikal		Vladivostok	
		Raw	Roasted	Raw	Roasted
С _4:0_	Butyric	0.1 ± 0.0^a^	0.1 ± 0.0^a^	–	–
С _6:0_	Caproic	0.8 ± 0.1^a^	0.5 ± 0.0^b^	–	–
С _8:0_	Caprylic	0.4 ± 0.0^a^	0.2 ± 0.0^b^	–	–
С _10:0_	Capric	0.1 ± 0.0^a^	0.1 ± 0.0^a^	–	–
С _12:0_	Lauric	0.2 ± 0.0^a^	0.1 ± 0.0^b^	–	–
С _14:0_	Myristic	0.2 ± 0.0^a^	0.1 ± 0.0^b^	–	–
С _14:1_	Myristoleic	0.1 ± 0.0	–	–	–
С _15:1_	Pentadecenoic	0.3 ± 0.0^a^	0.2 ± 0.0^b^	–	–
С _16:0_	Palmitic	4.1 ± 0.1^a^	4.2 ± 0.1^a^	7.6 ± 0.2^b^	8.0 ± 0.3^c^
С _16:1_	Palmitoleic	0.4 ± 0.0^a^	0.2 ± 0.0^b^	0.2 ± 0.0^b^	0.1 ± 0.0^c^
С _17:0_	Margaric	0.1 ± 0.0^a^	0.1 ± 0.0^a^	0.1 ± 0.0^a^	0.1 ± 0.0^a^
С _17:1_	Heptadecenoic	0.2 ± 0.0^a^	0.1 ± 0.0^b^	0.2 ± 0.0^a^	0.1 ± 0.0^b^
С _18:0_	Stearic	2.4 ± 0.2^a^	2.4 ± 0.1^a^	3.6 ± 0.1^b^	3.9 ± 0.2^c^
С _18:1_	Oleic	23.8 ± 0.4^а^	23.7 ± 0.2^а^	32.5 ± 0.4^b^	34.9 ± 0.6^c^
С _18:2_ trans	Linolelaidic	2.0 ± 0.0^a^	2.0 ± 0.1^a^	–	2.3 ± 0.1^b^
С _18:2_	Linoleic	42.5 ± 0.4^a^	43.5 ± 0.6^b^	40.7 ± 0.2^c^	37.5 ± 0.2^d^
С _18:3_	Pinolenic	18.0 ± 0.2^а^	18.4 ± 0.3^b^	10.6 ± 0.2^c^	8.6 ± 0.1^d^
С _18:3_	γ-Linolenic	0.3 ± 0.0^a^	0.2 ± 0.0^b^	0.2 ± 0.0^b^	0.1 ± 0.0^c^
С _18:3_	α-Linolenic	0.3 ± 0.0^a^	0.2 ± 0.0^b^	–	–
С _20:0_	Arachidic	0.3 ± 0.0^a^	0.3 ± 0.0^a^	0.6 ± 0.1^b^	0.6 ± 0.0^b^
С _20:1_	Eicosenoic	1.3 ± 0.1^a^	1.3 ± 0.0^a^	1.7 ± 0.1^b^	1.9 ± 0.2^b^
С _20:2_	5,11-Eicosadienoic	0.2 ± 0.0^a^	0.2 ± 0.0^a^	0.2 ± 0.0^a^	0.2 ± 0.0^a^
С _20:2_	*cis*-11,14- Eicosadienoic	0.6 ± 0.0^a^	0.6 ± 0.1^a^	0.7 ± 0.0^a^	0.7 ± 0.1^a^
С _20:3_	Sciadonic	1.1 ± 0.1^a^	1.1 ± 0.2^a^	0.8 ± 0.0^b^	0.8 ± 0.0^b^
С _20:3_ (n-6)	Dihomo-gamma-linolenic	0.1 ± 0.0^a^	0.1 ± 0.0^a^	0.1 ± 0.0^a^	0.1 ± 0.0^a^
С_22:0_	Behenic	0.1 ± 0.0^a^	0.1 ± 0.0^a^	0.2 ± 0.0^b^	0.1 ± 0.0^a^

* Different small letters in a row depict significant differences in the results (p < 0.05).

All the results were performed in triplicates (n = 3).

Fatty acid composition includes saturated and unsaturated fatty acids, which are very important for human metabolism. Unsaturated fatty acids are more specific when it comes to pine oils, and in turn, the quantity of saturated fatty acids (palmitic acid C16:0 and stearic acid C18:0) is significantly lower than monounsaturated fatty acid for investigated raw and roasted nuts from Vladivostok and Baikal.

According to Nergiz & Dönmez [34], the rate of unsaturated acids, especially linoleic and oleic, predominates. Wolff & Bayard [32] report the presence of pinolenic acid, which is typical for several species of pine nuts. They reported that lowest amount of this fatty acid was observed in the oil from *Pinus pinea* (0.35 %) and the highest was in *Pinus griffithii* in which its content could reach 21.78 %. In the investigated raw and roasted nuts from Baikal and Vladivostok, the acids identified are mainly linoleic acid (C18:2), oleic acid (C18:1), and pinoleic acid (C18:3). Their quantities differ from each other. The palmitic acid is about 4.0 % for nuts from Baikal, while the content for nuts from Vladivostok is twice as much. The data for nuts from Baikal matches with Meshgi & Asadi-Gharneh [43], where it is near 3.4 %. Oleic acid (18:1) content was close to 24 % for raw and roasted from Baikal and close to 35 % for raw and roasted from Vladivostok. The same tendency can be observed for the rest of the determined unsaturated fatty acids – linoleic and pinolenic. The results demonstrate that the roasting does not substantially change the fatty acid composition. The other determined fatty acids are in quantities lower than 1.0 %.

**Fig. 1:**
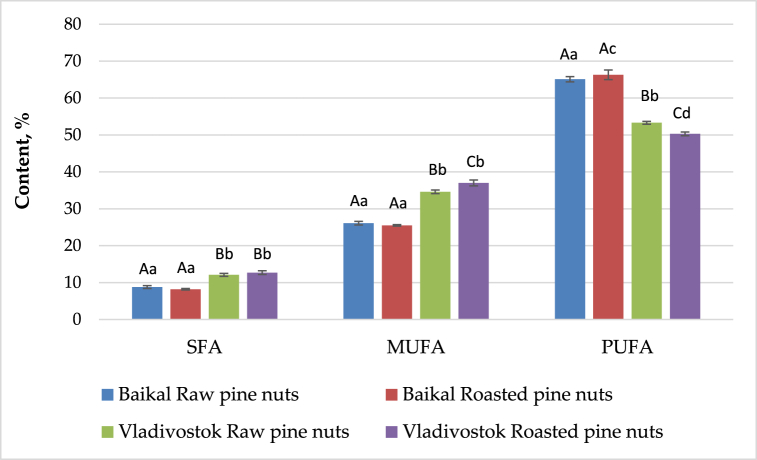
Content of saturated (SFA), unsaturated (UFA), mono- (MUFA) and polyunsaturated (PUFA) fatty acids of the raw and roasted pine nut oils * Different small letters depict significant differences in the results for raw/roasted nuts from different regions (p < 0.05); Different capital letters depict significant differences in the results for the nuts from same region, and differences between raw and roasted ones. All the results were performed in triplicates (n = 3).

The content of saturated (SFA), unsaturated (UFA), mono- (MUFA), and polyunsaturated (PUFA) fatty acids of the examined raw and roasted pine nut oils is given in Fig. 1.

No considerable differences are observed in the content of saturated, mono- and polyunsaturated fatty acids of the seed oil from Baikal after roasting. In contrast, in the seed oil from Vladivostok, a slight increase in the levels of monounsaturated fatty acids are observed, and a decrease in the content of polyunsaturated ones after the thermal treatment. Both raw and roasted nuts from Baikal are established to have higher content of PUFA than in the nuts from Vladivostok. These fatty acids are proved to possess some health benefits on the human body such as reducing the level of LDL cholesterol and respectively preventing from cardiovascular incidents.

The individual composition of the sterol, tocopherol, and phospholipid fraction is established. The content of individual bioactive compounds of raw and roasted pine nut oils is presented in Table 5.

**Table 5:** Sterol, tocopherol and phospholipid composition of raw and roasted pine nut oils.

Components, %	Baikal		Vladivostok	
	Raw	Roasted	Raw	Roasted
Sterols				
Cholesterol	5.8 ± 0.2^a^	4.0 ± 0.1^b^	5.9 ± 0.3^a^	5.9 ± 0.1^a^
Brassicasterol	1.3 ± 0.1^a^	2.8 ± 0.2^b^	4.8 ± 0.1^c^	6.4 ± 0.2^d^
Campesterol	2.9 ± 0.2^a^	3.7 ± 0.2^b^	10.7 ± 0.2^c^	5.8 ± 0.1^d^
Stigmasterol	1.2 ± 0.1^a^	1.5 ± 0.2^a^	4.0 ± 0.2^b^	1.3 ± 0.2^a^
Δ^7^ - Campesterol	3.1 ± 0.2^a^	1.0 ± 0.1^b^	2.0 ± 0.2^c^	–
β - Sitosterol	85.7 ± 0.4^a^	86.6 ± 0.6^a^	71.2 ± 0.3^b^	80.6 ± 0.5^c^
Δ^5^ - Avenasterol	–	–	1.0 ± 0.1	–
Δ^7^ - Stigmasterol	–	0.4 ± 0.0^a^	0.3 ± 0.0^b^	–
Δ^7^ - Avenasterol	–	–	0.1 ± 0.0	–
Tocopherols				
α-Tocopherol	55.0 ± 0.6^a^	53.8 ± 0.2^b^	47.0 ± 0.4^c^	48.0 ± 0.3^d^
γ-Tocopherol	45.0 ± 0.4^a^	46.2 ± 0.2^b^	53.0 ± 0.5^c^	52.0 ± 0.2^d^
Phospholipids				
Phosphatidylcholine	35.5 ± 0.8^a^	49.3 ± 1.1^b^	37.4 ± 0.6^c^	42.5 ± 0.4^d^
Phosphatidylinositol	26.6 ± 0.5^a^	14.9 ± 0.3^b^	4.5 ± 0.1^c^	6.4 ± 0.4^d^
Phosphatidylethanolamine	9.4 ± 0.2^a^	8.2 ± 0.2^b^	5.4 ± 0.2^c^	1.3 ± 0.2^d^
Phosphatidylserine	3.5 ± 0.1^ab^	3.2 ± 0.2^a^	4.9 ± 0.2^c^	3.7 ± 0.2^b^
Phosphatidic acids	9.5 ± 0.4^a^	6.8 ± 0.2^b^	1.1 ± 0.1^c^	1.5 ± 0.1^d^
Sphingomyelin	4.3 ± 0.2^a^	4.5 ± 0.4^a^	0.4 ± 0.1^b^	0.3 ± 0.1^b^
Diphosphatidylglycerol (Cardiolipin)	3.5 ± 0.1^a^	3.4 ± 0.2^a^	12.3 ± 0.3^b^	8.3 ± 0.1^c^
Lysophosphatidylcholine	3.5 ± 0.2^a^	4.7 ± 0.4^b^	19.3 ± 0.3^c^	20.0 ± 0.5^c^
Lysophosphatidylethanol-amine	4.2 ± 0.1^a^	5.0 ± 0.3^b^	14.7 ± 0.4^c^	16.0 ± 0.6^d^

* Different small letters in a row depict significant differences in the results (p < 0.05).

All the results were performed in triplicates (n = 3).

As the significant components, the individual sterol composition is dominated by β – sitosterol, campesterol, cholesterol, and brassicasterol. The content of β – sitosterol for pine nuts from Baikal and Vladivostok agrees with other authors [44], followed by campesterol for Vladivostok. In contrast, the content of cholesterol from Baikal is more than campesterol. No substantial difference is observed in the quantity of β – sitosterol after roasting pine nuts from Baikal – 85.7 % (raw) and 86.6 % (roasted). The heat treatment affects the amount of β – sitosterol for Vladivostok pine nut oil as it increases significantly, and the content for roasted nuts is 80.6 %. The increased quantity of brassicasterol is also determined after roasting in both oils from both regions.

There are considerable differences between the amount of campesterol in oils from Baikal and Vladivostok. The content of campesterol increases significantly after roasting nuts from Baikal – 2.9 % (raw) and 3.7 % (roasted) and decreases drastically after heat treatment for pine nuts from Vladivostok – 10.7 % (raw) and 5.8 % (roasted). According to Vecka et al. [44], the content of campesterol is 5.0 %, which is close to that of roasted nuts from Vladivostok. The cholesterol is present in equal amounts before and after roasting the nuts from Vladivostok and significantly decreases after heat treatment for the ones from Baikal. The presence of cholesterol results from the same biosynthetic pathway as plant sterols (i.e., via cycloartenol as a key intermediate).

Tocopherols (known as Vitamin E) are excellent natural antioxidants and important biologically active substances. The oils are especially rich in two types of tocopherols (α- and γ-tocopherols). The content of α-tocopherol is higher for the oils from Baikal, while in the oils from Vladivostok, γ-tocopherol predominates. The content of γ-tocopherol for the investigated oil (Baikal) increases after roasting, while α-tocopherol content decreases after the same process (p < 0.05). The tendency for Vladivostok oils is the opposite - α-tocopherol content increases while γ-tocopherol decreases.

All phospholipids typical for glyceride plant oils are observed in pine nut oils from Baikal and Vladivostok. The primary phospholipid compound in the raw seeds from the two regions is phosphatidylcholine (35.5 and 37.4 %). The second highest component in the raw seeds from Baikal is phosphatidylinositol (26.6 %), followed by phosphatidylethanolamine (9.4 %) and phosphatidic acids (9.5 %). The raw seeds from Vladivostok differ in the phospholipid composition – the following phospholipids identified are also in high amounts: lysophosphatidylcholine (19.3 %), lysophosphatidylethanolamine (14.7 %) and diphosphatidylglycerol (12.3 %).

Notable differences are observed in the major phospholipid components' content after roasting the seeds (p < 0.05). The amounts of phosphatidylcholine and lysophosphatidylethanolamine increase substantially in the seeds from both regions, while a decrease is observed in phosphatidylethanolamine content. The quantity of phosphatidylinositol in the seeds from Baikal is reduced almost twice after thermal treatment, while in those from Vladivostok, it slightly increases.

## 4 Conclusions

This study investigated the influence and changes in chemical and lipid composition and the oxidative stability of two species of pine oils isolated from raw and roasted nuts due to heat treatment at high temperature (180 °C). The roasting process led to the most significant changes in peroxide value and some unsaturated fatty acids for oils from Vladivostok and small changes in the chemical and lipid composition of oils from Baikal. That means the raw and roasted pine nuts can be consumed regardless of the influence of high temperatures. They have a high energy value and high protein content, and they have excellent health benefits.

## Funding

This research received no external funding.

## Data availability statement

The data presented in this study are available on request from the corresponding author.

## CRediT authorship contribution statement

**Olga Teneva:** Writing – original draft, Validation, Supervision, Methodology, Investigation, Formal analysis, Data curation, Conceptualization. **Zhana Petkova:** Writing – review & editing, Validation, Software, Methodology, Investigation, Formal analysis, Data curation. **Nikolay Toshev:** Writing – review & editing, Resources, Project administration. **Nikolay Solakov:** Software. **Kamelia Loginovska:** Validation, Formal analysis. **Yurii Platov:** Writing – review & editing, Resources.

## Declaration of competing interest

The authors declare that they have no known competing financial interests or personal relationships that could have appeared to influence the work reported in this paper.

## References

[1] FAO (2003). Food and agriculture organization of the United Nations. Food energy – methods of analysis and conversion factors. FAO Food and Nutrition Paper, Report of a Technical Workshop.

[2] Lutz M., Álvarez K., Loewe V. (2017). Chemical composition of pine nut (Pinus pinea L.) grown in three geographical macrozones in Chile. CYTA - J. Food.

[3] Yang X., Zhang H., Zhang Y., Zhao H., Dong A., Xu D. (2010). Analysis of the essential oils of pine cones of Pinus koraiensis Steb. Et Zucc. and P. sylvestris L. from China. J. Essent. Oil Res..

[4] Matthaus B., Özcan M.M. (2013). Fatty acid, tocopherol and sterol contents of forest pine seed oil. Asian J. Org. Chem..

[5] INC (2017). Nuts and Dried Fruits Statistical Yearbook.

[6] USDA Foreign Agricultural Service (2011). https://apps.fas.usda.gov/newgainapi/api/Report/DownloadReportByFileName?fileName=Russia+Going+Nuts+Over+Almonds_St.+Petersburg_Russian+Federation_4-20-2011.pdf.

[7] O'Neil C.E., Keast D.R., Fulgoni V.L., Nicklas T.A. (2010). Tree nut consumption improves nutrient intake and diet quality in US adults: an analysis of National Health and Nutrition Examination Survey (NHANES) 1999-2004. Asia Pac. J. Clin. Nutr..

[8] Higgs J. (2003). The beneficial role of peanuts in the diet – Part 2. Nutr. Food Sci..

[9] Alper C.M., Mattes R.D. (2003). Peanut consumption improves indices of cardiovascular disease risk in healthy adults. J. Am. Coll. Nutr..

[10] Stintzing F.C., Hoffmann M., Carle R. (2006). Thermal degradation kinetics of isoflavone aglycones from soy and red clover. Mol. Nutr. Food Res..

[11] Rizki H., Kzaiber F., Elharfi M., Ennahli S., Hanine H. (2015). Effects of roasting temperature and time on the physicochemical properties of sesame (Sesamum indicum L.) seeds. Int. J. Innovat. Appl. Stud..

[12] Hosseini Bai S., Darby I., Nevenimo T., Hannet G., Hannet D., Poienou M. (2017). Effects of roasting on kernel peroxide value, free fatty acid, fatty acid composition and crude protein content. PLoS One.

[13] An J., Adelina N.M., Zhang L., Zhao Y. (2022). Effect of roasting pre‐treatment of two grafted pine nuts (*Pinus koraiensis*) on yield, color, chemical compositions, antioxidant activity, and oxidative stability of the oil. J. Food Process. Preserv..

[14] AOAC (Association of Official Analytical Chemist) (2016).

[15] ISO 659 (2014).

[16] ISO 3960 (2007). Determination of Peroxide Value.

[17] ISO 660 (2009).

[18] AOCS (American Oil Chemists Society) (1999).

[19] ISO 3656 (2011). Determination of Ultraviolet Absorbance Expressed as Specific UV Extinction.

[20] ISO 6886 (2006). Determination of Oxidative Stability (Accelerated Oxidation Test).

[21] Brand-Williams W., Cuvelier M.E., Berset C.L.W.T. (1995). Use of a free radical method to evaluate antioxidant activity. LWT--Food Sci. Technol..

[22] ISO 12966-1 (2014). Gas Chromatography of Fatty Acid Methyl Esters – Part 1: Guidelines on Modern Gas Chromatography of Fatty Acid Methyl Esters.

[23] ISO 12966-2 (2011).

[24] ISO 18609 (2000). Determination of Unsaponifiable Matter.

[25] Ivanov S., Bitcheva P., Konova B. (1972). Méthode de détermination chromatographyque et colorimétrique des phytosterols dans les huiles végétales et les concentres steroliques. Rev. Fr. Corps Gras.

[26] ISO 12228-1 (2014). Determination of Individual and Total Sterols Contents. Gas Chromatographic Method.

[27] ISO 9936 (2016). Determination of Tocopherol and Tocotrienol Contents by High-Performance Liquid Chromatography.

[28] Folch J., Lees M., Sloane-Stanley G.H. (1957). A simple method for isolation and purification of total lipids from animal tissues. J. Biol. Chem..

[29] Schneiter R., Daum G., Xiao W. (2006). Yeast Protocol: Second Edition, Methods in Molecular Biology, 313.

[30] ISO 10540-1 (2014).

[31] Valero-Galván J., Reyna-González M., Chico-Romero P.A., Martínez-Ruiz N.D.R., Núñez-Gastélum J.A. (2019). Seed characteristics and nutritional composition of pine nut from five populations of *P. cembroides* from the states of Hidalgo and Chihuahua, Mexico. Molecules.

[32] Wolff R.L., Bayard C.C. (1995). Fatty acid composition of some pine seed oils. J. Am. Oil Chem. Soc..

[33] Zuleta A. Weisstaub, Giacomino S., Dyner L., Loewe Muñoz V., Río R. Del (2018). An ancient crop revisited: chemical composition of Mediterranean pine nuts grown in six countries. Ital. J. Food Sci..

[34] Nergiz C., Dönmez I. (2004). Chemical composition and nutritive value of *Pinus pinea* L. seeds. Food Chem..

[35] Lixia H.O.U., Cuicui L.I., Jihong Q.I.U. (2018). Comparison of the physicochemical characteristics of *Pinus koraiensis* L. nut oils from different extraction technologies. Grain and Oil Science and Technology.

[36] Marmesat S., Velasco J., Ruiz-Méndez M.V., Dobarganes M.C. (2006). Oxidative quality of commercial fried nuts: evaluation of a surface and an internal lipid fraction. Grasas Aceites.

[37] Olatidoye O.P., Shittu T.A., Awonorin S.O., Ajisegiri E.S. (2020). Influence of roasting conditions on physicochemical and fatty acid profile ofraw and roasted cashew kernel (*Anacardium occidentale*) grown in Nigeria. Hrvat. čas. za prehrambenu tehnol. biotehnol. Nutr..

[38] Popov A., Yanishlieva N. (1976).

[39] El-Labban A.A. (2022). Impact of roasting process on the quality of flaxseeds. Egyptian Journal of Applied Science.

[40] Stan Codex, Codex Alimentarius Commission (2006).

[41] Durmaz G., Gökmen V. (2010). Impacts of roasting oily seeds and nuts on their extracted oils. Lipid Technol..

[42] Chen J., Hong Z.T., Liu G.Q., H Wang Y. (2015). Effect of microwave roasting on the quality and volatile compounds of sunflower oil. Modern Food Science and Technology.

[43] Meshgi V., Asadi-Gharneh H.A. (2019). Oil content and fatty acid profile of some pine nuts species (Pinus spp.). J. Nuts.

[44] Vecka M., Staňková B., Kutová S., Tomášová P., Tvrzická E., Žák A. (2019). Comprehensive sterol and fatty acid analysis in nineteen nuts, seeds, and kernel. SN Appl. Sci..

